# Giant nontraumatic intradiploic arachnoid cyst in a young male*

**DOI:** 10.1590/0100-3984.2013.0022

**Published:** 2016

**Authors:** Rajesh Sharma, Puneet Gupta, Manik Mahajan, Poonam Sharma, Anchal Gupta, Arti Khurana

**Affiliations:** 1 MD, Department of Radiodiagnosis and Imaging, ASCOMS Hospital, Sidhra, Jammu (J&K), India.; 2 MD, Department of Radiodiagnosis and Imaging, Lady Hardinge Medical College, New Delhi, India.; 3 MD, Department of Pathology, GMC, Jammu (J&K), India.; 4 MD, Department of Radiodiagnosis and Imaging, GMC, Jammu (J&K), India.

**Keywords:** Arachnoid cysts, Cerebrospinal fluid, Headache/diagnosis

## Abstract

Intradiploic arachnoid cysts have scarcely been reported in the literature, most reported cases being secondary to trauma. Nontraumatic arachnoid cysts are quite rare and have been reported mostly in adults. Here, we report the case of a 16-year-old male presenting with a slowly growing mass in the occipital region and intermittent headaches. On the basis of the findings of X-rays, computed tomography scans, and magnetic resonance imaging scans of the head, the mass was diagnosed as a giant intradiploic arachnoid cyst.

## Abstract (pt)

Cistos aracnóideos intradiploicos foram raramente relatados na literatura, sendo a maior parte desses casos decorrente de traumas. Cistos aracnóideos não traumáticos são muito raros e têm sido relatados principalmente em adultos. Aqui, relatamos o caso de um jovem de 16 anos de idade apresentando uma massa de crescimento lento na região occipital e dores de cabeça intermitentes. Com base nos resultados de radiografia, tomografia computadorizada e ressonância magnética da cabeça, a massa foi diagnosticada como um cisto aracnóideo intradiploico gigante.

## INTRODUCTION

Arachnoid cysts are benign developmental abnormalities of the arachnoid membrane that can occur anywhere along the cerebrospinal axis. They are rare lesions, accounting for only 1% of all intracranial space-occupying lesions^(1)^. Intraosseous cerebrospinal fluid (CSF) cysts are uncommon lesions of the cranium. The terms arachnoid cyst, leptomeningeal cyst, and CSF fistula have been used interchangeably to describe these cysts, which constitute a rare type of growing skull fracture and occur following severe head injury in individuals ≤ 3 years of age. Nontraumatic CSF-containing cystic lesions of the skull are extremely rare and are presumed to be congenital.

Here, we describe the X-ray, computed tomography (CT), and magnetic resonance imaging (MRI) findings of a giant nontraumatic intradiploic arachnoid cyst in a 16-year-old male. Nontraumatic intradiploic arachnoid cyst is rare, only 15 cases having been reported to date^(2)^, most in individuals over 50 years of age. To our knowledge, this is the first case of a giant intradiploic arachnoid cyst in a young male.

## CASE REPORT

**Figure 1:**
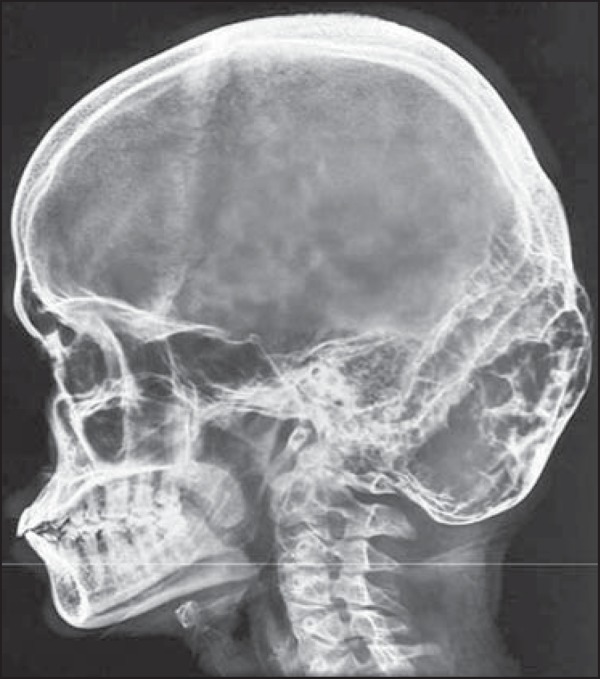
Lateral X-ray of the skull, showing a large, ill-defined, well-demarcated, expansile, multiloculated osteolytic lesion in the occipital bone.

**Figure 2:**
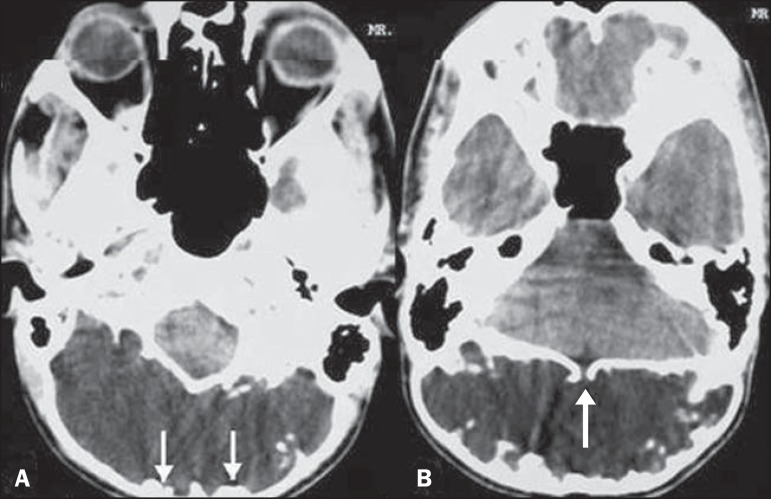
**A:** Non-contrast-enhanced CT scan of the head showing a large CSF-density lesion measuring approximately 9 cm × 5 cm, together with expansion, thinning, and marked scalloping of the occipital bone (arrows). **B:** Non-contrast-enhanced CT scan of the head showing a well-defined bony defect with smooth margins in the occipital bone (arrow). Note the communication with the subarachnoid space.

**Figure 3:**
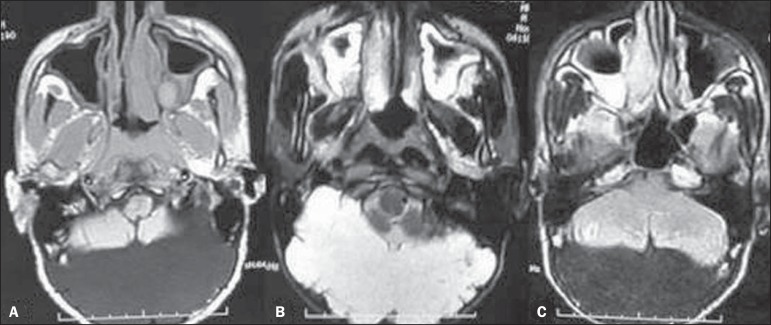
**A,B:** Axial T1- and T2-weighted MRI scans showing near-total replacement of the posterior fossa with a CSF-intensity lesion, with anterior displacement of the cerebellar hemispheres. **C:** FLAIR images showing complete suppression of the signal in the lesion.

A 16-year-old male presented with an 8- to 9-year history of a painless, slow-growing swelling on the scalp and intermittent headache. He had no history of head trauma. Physical examination revealed that, in the occipital region, there was a large, ill-defined, swelling that was non-tender, bony, and hard. For further evaluation, an X-ray of the skull and a CT scan of the head were ordered. The X-ray revealed a large, ill-defined, well-demarcated, expansile, multiloculated osteolytic lesion in the occipital bone (Figure 1). The CT scan, which was obtained without contrast, revealed a large CSF-density lesion measuring approximately 9 cm × 5 cm, with expansion, thinning, and marked scalloping of the occipital bone (Figure 2A). A well-defined bony defect with smooth margins was seen in the occipital bone, through which there was anterior communication between the lesion and the intracranial subarachnoid space (Figure 2B). The differential diagnosis included arachnoid cyst, epidermoid cyst, and aneurysmal bone cyst. An MRI scan of the brain was performed for further investigation. The MRI (T1- and T2-weighted sequences) revealed that the posterior fossa had been almost entirely replaced with a CSF-density lesion, with anterior displacement of the cerebellar hemispheres (Figures 3A and 3B). Fluid-attenuated inversion recovery (FLAIR) images revealed complete suppression of the signal (Figure 3C). No fluid levels were demonstrated.

On the basis of the CT and MRI findings, a diagnosis of giant intradiploic arachnoid cyst was made. The patient was advised to undergo exploratory surgery but refused and was therefore discharged with a prescription for analgesics. At one month of follow-up, he was asymptomatic.

## DISCUSSION

The terms arachnoid cyst, leptomeningeal cyst, and CSF fistula have been used interchangeably. They are described as benign intradiploic cystic lesions of the cranium. The characteristic findings in these lesions are an intact outer table and CSF-filled cyst which communicates with the subarachnoid space intracranially through a defect in the inner table. These cysts are not entirely between the two tables of the skull, because they almost always feature a communication with the intracranial subarachnoid space^(3)^.

Intraosseous CSF-containing cysts are typically seen in association with post-traumatic growing skull fracture^(4,5)^. There is herniation of the arachnoid membrane through the bony fissure, caused by pulsatile CSF flow^(6,7)^. Over the course of several years, continuous pulsations erode the edges of the fractured inner table and lead to the development of intraosseous cysts. The intradiploic cyst thus formed elevates the outer table and flattens the inner table.

Nontraumatic CSF cysts are comparatively much rarer than are those occurring after head trauma. Nontraumatic intradiploic arachnoid cysts develop as a diverticulum of the arachnoid membrane through small defects in the dura mater. The most common location is the suboccipital midline region. Other sites include the frontotemporal region and anterior cranial fossa. Although most patients are asymptomatic and are diagnosed incidentally, some present clinically with progressive swelling, with or without headache.

Intradiploic arachnoid cyst is a rare osteolytic lesion of the skull. It is characterized by multiple, symmetrical, well-demarcated occipital osteolytic lesions on routine skull radiography^(8)^. On CT scans, it presents as an intradiploic cystic lesion, in a location associated with bone remodeling and communicating with the cistern magna. On T1- and T2-weighted MRI scans with complete suppression on FLAIR, as well as on diffusion-weighted images, a fluid-intensity lesion can be seen. A definite communication with the subarachnoid space can be demonstrated in the majority of cases.

Nontraumatic intradiploic arachnoid cysts are rare, only 15 cases having been reported to date. These cysts were first described in 1989 by Weinand et al.^(9)^, who characterized them as multiple, well-demarcated, osteolytic lesions, commonly found in the parasagittal occipital region. Due to rarity of the condition, its natural history remains unclear. The lesions are characteristically thin-walled and enlarge gradually, rendering the bone wall papery thin. Normal CSF pressure would not be enough to cause an expansile lesion in intact bone, and this process is therefore presumed to have started before the ossification of the cranium. In some cases, a small amount of dysplastic brain tissue has also been seen herniating, together with CSF, through the bony defect^(10)^.

The various differential diagnoses of CSF-containing, intraosseous, expansile cystic bony lesion of the brain include intraosseous epidermoid/dermoid cyst, aneurysmal bone cyst, fibrous dysplasia, eosinophilic granuloma, hydatid cyst, and malignant conditions such as plasmacytoma, myeloma, osteogenic sarcoma, and intraosseous meningioma. Eosinophilic granulomas and neoplasms are osteolytic rather than expansile lesions and their contents are not of CSF density. Fibrous dysplasia cystic lesions are usually quite small and multiple with attendant sclerosis of bone. The use of MRI, especially with fat suppression sequences, can help exclude epidermoid and dermoid cysts^(1)^.

The surgical management of these cysts includes excision of the pedicle and repair of small dural and bony defects. However, given the benign nature of the lesion, small asymptomatic lesions can be monitored radiologically^(10,11)^.
